# Belladonna in Salivation

**Published:** 1855-01

**Authors:** 


BELLADONNA IN SALIVATION.—A woman treated by mercury, internally and externally, for serous diarrhœa, was affected with profuse salivation. Dr. Erpenbeck treated this latter complaint with belladonna in divided doses, of two grains and a half, taken in emulsion every twenty-four hours. Next day the salivation had subsided, and the mouth was quite dry. On stopping the belladonna, the salivatiou returned, and again ceased when it was resumed. The author believes that after this fact and some similar ones, belladonna is the best treatment for salivation.—*Hanover Corresp. Blatt.*

